# Acellular Pertussis Vaccine Components: Today and Tomorrow

**DOI:** 10.3390/vaccines8020217

**Published:** 2020-05-13

**Authors:** Kalyan K. Dewan, Bodo Linz, Susan E. DeRocco, Eric T. Harvill

**Affiliations:** 1 Department of Infectious Diseases, College of Veterinary Medicine, University of Georgia, Athens, GA 30602, USA; kaldew@uga.edu (K.K.D.); bodo.linz@uga.edugmail.com (B.L.); 2 The Lockwood Group, Stamford, CT 06901, USA; susan@thelockwoodgrp.com

**Keywords:** pertussis, acellular pertussis vaccine, whole-cell pertussis vaccine, pertussis toxin, pertactin, filamentous hemagglutinin, fimbriae

## Abstract

Pertussis is a highly communicable acute respiratory infection caused by *Bordetella pertussis*. Immunity is not lifelong after natural infection or vaccination. Pertussis outbreaks occur cyclically worldwide and effective vaccination strategies are needed to control disease. Whole-cell pertussis (wP) vaccines became available in the 1940s but have been replaced in many countries with acellular pertussis (aP) vaccines. This review summarizes disease epidemiology before and after the introduction of wP and aP vaccines, discusses the rationale and clinical implications for antigen inclusion in aP vaccines, and provides an overview of novel vaccine strategies aimed at better combating pertussis in the future.

## 1. Introduction

Pertussis (whooping cough) is a highly communicable, acute respiratory infection caused by *Bordetella pertussis* [1,2]. Based on a model developed by the World Health Organization (WHO), the number of global pertussis cases in children <5 years of age in 2014 was estimated at >24.1 million with 160,700 deaths [3]. Transmission is believed to occur from person to person through aerosol droplets and/or fomites, and once in the respiratory tract, bacteria attach to ciliated epithelial cells and release toxins that lead to inflammation and the disruption of normal cell function [1,2]. The first phase (catarrhal) of disease has symptoms similar to those of the common cold, including runny nose, low-grade fever and mild cough [1]. The second stage (paroxysmal) is characterized by bursts of rapid coughing (paroxysm), often followed by a characteristic high-pitched “whoop” that occurs as air reenters the lungs when patients gasp to breathe. Recovery (convalescence) from the disease is a gradual process, with the episodes of coughing subsiding over the course of months. Pertussis tends to be more severe in infants and young children compared with older children, adolescents and adults, owing to a qualitative difference in the immune system because of a lag in thymic maturation; even in adults, immunity is not permanent as evidenced by the reemergence of disease after prior infection or previous vaccination [1,4].

Whole-cell pertussis (wP) vaccines, composed of heat-killed *B. pertussis* containing many bacterial antigens, were approved for use in the 1940s [5,6]. Over the past several decades, the inclusion of pertussis vaccines in global immunization programs of infants and young children has effectively reduced the incidence of pertussis in these age groups [7]. In the United States (US), inclusion of wP vaccines into infant immunization programs resulted in a decrease in pertussis cases, as evidenced by a high of ~270,000 reported cases in peak years before vaccine introduction to a nadir of 1010 reported cases in 1976 (~99% reduction) [8]. In the face of waning disease, injection-site and systemic reactions related to wP vaccines became of greater concern, leading to the development of considerably less reactogenic acellular pertussis (aP) vaccines [9,10]. Composed of up to 5 purified *B. pertussis* antigens, aP vaccines have fewer distinct individual antigens compared with wP vaccines and are used in most industrialized countries [7], with immunogenicity and safety studies underway or completed in a number of emerging countries including Gambia (ClinicalTrials.gov identifier: NCT03606096), Argentina and India, among others [11,12,13]. Numerous formulations have been developed that differ in the number, type and quantity of antigens; purification and detoxification methods; and adjuvant and excipient type [9]. Head-to-head clinical studies of aP and wP vaccines have confirmed the favorable efficacy and safety profiles of aP vaccines [14,15], and years of real-world use have solidified their utility in routine immunization schedules [7]. Despite a large body of clinical and epidemiological data, evaluating the true immunological impact of individual aP vaccine components and overall vaccine efficacy has been challenging and is compounded by the lack of a single, universally accepted correlate of protection.

Despite the routine use of vaccines, increases in pertussis reports have been observed worldwide, including in countries with typically high vaccination coverage. However, the observed resurgence of pertussis is not universal, and there is substantial variability across regions and some countries, with large fluctuations in cases over time [16,17]. Multiple factors have influenced the pertussis resurgence, including improved detection methodology, awareness and reporting; antigenic shifts; increased transmission by asymptomatic individuals; and waning immunity and lack of natural boosting [16]. The uptick in pertussis was temporally associated with the switch from wP to aP vaccines in some countries, such as the United Kingdom (UK) and Spain, while increasing trends were noted before the switch from wP to aP vaccines in Australia, Bulgaria, Finland, Israel, the Netherlands, Poland and the US [16,17]. Moreover, increased incidence has also been reported in countries that immunize with wP vaccines for the primary infant series [17]. Pertussis has increased in older children, adolescents, adults and infants too young to be vaccinated. From an immunological perspective, this is not unexpected after successful pertussis vaccination programs in young children in which decreased circulation of pertussis within the population (due to vaccination) limits exposure and boosting that would normally occur after repeated, natural exposures in an environment where the bacterium previously flourished [8]. Women of childbearing age also have decreased exposure due to reductions in circulating pertussis, which reduces the production and placental transfer of antipertussis antibodies and creates a gap in protection among infants too young to be vaccinated [8]. Variable antigenicity of different wP and aP formulations and interpatient variability in the immune response adds an additional layer of complexity [16].

Due to the cyclic nature of pertussis outbreaks (~3–4 years) and the lack of lifelong immunity after pertussis vaccination and natural infection, immunization strategies are needed beyond the standard booster dosing recommended following the infant series. Recommendations from the US Centers for Disease Control and Prevention (CDC) include the following strategies to address ongoing pertussis concerns and complement 5-dose vaccination schedules for infants and children 2 months through 6 years of age: a universal adolescent vaccination at 11–18 years of age; vaccination for those ≥19 years of age who were not vaccinated during adolescence; and vaccination during each pregnancy, optimally from 27 through 36 weeks’ gestation [18]. Pertussis is less contagious in partially vaccinated compared with unvaccinated individuals, which underscores the importance of vaccination for older children, adolescents and adults for maintaining overall disease control [19].

Additional strategies are also needed for infants too young for routine vaccination. In response to a spike in pertussis-related illness and deaths in unimmunized infants <3 months of age in the UK in 2011, a vaccination program during pregnancy was implemented in 2012 that targeted women at ≥28 weeks of gestation [20]. Over 3 years of this program, pregnancy vaccination was estimated to be 91% (95% CI: 88%, 94%) effective at preventing pertussis in infants <3 months of age. The effectiveness of the 5-component aP vaccine that was exclusively used for pregnancy vaccination during the first 21 months of the program was 93% (95% CI: 89%, 95%), while that of the 3-component vaccine used thereafter was 88% (95% CI: 79%, 93%) [20].

The purpose of this review is to discuss the properties and value of antigens included in multicomponent aP vaccines. Limited data regarding correlates of protection are summarized, and an overview of novel strategies aimed at potentially improving longer-term protection, either through the use of new vaccines or modifications to existing aP vaccines, is provided.

## 2. Pertussis Antigens in Acellular Vaccines

Up to 5 bacterial antigens are included in the currently licensed pediatric and adult aP vaccines: pertussis toxoid (PT) and up to 4 adhesion proteins of filamentous hemagglutinin (FHA), pertactin (PRN) and fimbriae (FIM) types 2 and 3 (FIM2/3); selection of these proteins as vaccine antigens is supported by their roles in disease pathology and utility as immunological targets [18].

### 2.1. Pertussis Toxin

Pertussis toxin is a key virulence factor that is specific for *B. pertussis* and responsible for most systemic symptoms associated with pertussis disease [21,22]. Functionally, pertussis toxin disrupts host cell signaling through G-protein–coupled receptors and alters glucose metabolic pathways leading to hyperinsulinemia, dampened immunity, and increased bacterial load [23]. Murine models of pertussis have used wild-type and pertussis toxin-deficient strains to delineate the immunomodulatory effects of pertussis toxin that promote disease [21]. In alveolar tissue, pertussis toxin inhibits early neutrophil recruitment, induces a proinflammatory response that delays antibody-mediated clearance [24], suppresses adaptive immunity by reducing major histocompatibility complex class II and CD1a expression on monocytes [25,26], and is thought to promote coughing and transmission [21].

Before inclusion as a vaccine component, pertussis toxin must be detoxified, and the reagents and processes used to accomplish this have differential effects on immunogenicity. Chemical detoxification includes formaldehyde and glutaraldehyde, which are more destructive to antigenic epitopes compared with hydrogen peroxide, another chemical detoxicant [27]. Detoxification can also be achieved through genetic modification, which does not change the antigenic characteristics and may be immunologically superior to chemical treatment [15,27]; however, few approved vaccines use genetically modified pertussis toxin. Based on the detoxification method, different amounts of pertussis toxin are needed to achieve comparable immunogenicity, and although differences in the detoxification methods and quantities of pertussis toxin can complicate across-study interpretation, the overarching finding is that pertussis toxin is an essential vaccine antigen, and has been included in all currently licensed aP vaccines [27].

A diphtheria, tetanus and aP vaccine containing PT as the only pertussis antigen has been used in infant vaccination schedules in Denmark since 1997, and the burden of pertussis is comparable in Denmark to that in neighboring countries that use multicomponent aP vaccines [28]. In fully immunized infants, vaccine efficacy for a 1-component PT vaccine was approximately 80% and similar to that observed for 3- and 5-component aP vaccines [28]. These data are in accordance with a study conducted in Sweden in a similar patient population in which efficacy was 71% with a 1-component PT vaccine [29].

### 2.2. Filamentous Hemagglutinin

FHA is a highly expressed, immunogenic bacterial surface-associated and secreted protein that promotes adhesion to ciliated epithelium and phagocytosis by macrophages and polymorphonuclear leukocytes [22,30]. FHA may also suppress inflammatory pathways in the respiratory tract through stimulation of cytokines, particularly interleukin (IL)-6 and IL-10 [31]. In mouse models, antibodies to FHA were the least protective of all vaccine antigens against pertussis challenge, but did boost the protection conferred by PT [32]. In humans, FHA can augment the PT response, but this is not consistent across studies [33,34], and better protection is achieved when FHA is administered with an adjuvant that stimulates mucosal immunity [35].

In a clinical study conducted in Sweden in 1986, protective efficacy against culture-confirmed pertussis was 69% for a 2-component vaccine containing PT and FHA when vaccines were administered on a 2-dose schedule compared with 54% for a 1-component PT vaccine [33]. The difference between vaccines was not statistically significant, and the overall findings did not demonstrate that FHA improved protection provided by 1-component vaccines [33]. In contrast, another study in humans found that the relative efficacy of a Japanese monovalent PT vaccine was significantly less than that seen from a combined PT-FHA vaccine during 3 years of follow-up [36].

### 2.3. Pertactin

PRN is a bacterial surface protein associated with adhesion to the respiratory epithelial cells [30,37,38]. In mouse models, PRN contributes to pathogenesis by inhibiting neutrophil-mediated clearance in the lower respiratory tract resulting in a sustained inflammatory response [39]. The role of PRN in pathogenesis in humans has not been fully elucidated and has been further called into question based on the emergence of PRN-deficient strains worldwide; these strains comprise >70% of isolates in the US and Australia [40].

Clinical study data suggest a role for PRN in disease protection. In a randomized study of approximately 83,000 infants receiving a 3-dose primary series with a 2-, 3- and 5-component aP vaccine, symptomatic culture-confirmed pertussis was more frequent in those receiving an experimental 2-component vaccine, leading to the speculation that PRN may confer additional protection over PT and FHA alone [41]. In 2 systematic reviews, aP vaccines with at least 3 components (PT, FHA and PRN) had higher efficacy against pertussis in the clinical trial setting compared with 1- and 2-component vaccines [42,43]. However, in one of these meta-analyses, removal of data for the 2-component vaccine (which was never marketed) resulted in no differences between aP vaccines with ≥3 components and those with 1 component [43,44].

### 2.4. Fimbriae Types 2 and 3

FIM2 and FIM3 are long, filamentous, surface proteins that mediate bacterial adhesion and suppression of the inflammatory response [30,45]. FIM2 and FIM3 are transcribed from independent loci but have similar transcriptional regulatory elements, and *B. pertussis* strains can predominantly express FIM2, FIM3 or both [46,47]. In vitro, antibodies to FIM2 and FIM3 inhibited bacterial attachment to Vero cells [45] and in a tracheal organ culture model, mutant strains lacking FIM had reduced adherence compared with a wild-type strain [48]. In a study using postvaccination human sera, antibodies specific to FIM, but not to the other aP vaccine antigens, prevented bacterial attachment to ciliated epithelia in vitro and induced most agglutination in postvaccination and postinfection sera that contributed to reduced bacterial attachment [49].

Antibodies to FIM may be an important factor for protection against pertussis. In mice receiving a booster after initial vaccination, antibodies against FIM2/3 increased protection and reduced colonization after intranasal challenge, with efficacy increasing in a dose-dependent manner and without additional reactogenicity [50]. FIM antibodies may bolster the immunity to pertussis by promoting opsonophagocytosis to a greater degree than other aP vaccine antigens [45], but additional studies in this area are needed. The timing of exposure to FIM is also important, with greater antibody responses elicited when the initial vaccine contains FIM; antibody responses to FIM2/3 after infection were higher in those who received a 5-component or wP vaccine compared with a 3-component vaccine [51].

Clinical study data support the role of FIM in inducing protection against pertussis (Table 1). In a study in Swedish children with household exposure to pertussis who had been vaccinated with a 2- or 5-component aP or wP vaccine, only the 5-component vaccine containing FIM2/3 induced acceptable efficacy (75.4% against pertussis with ≥21 days of paroxysmal cough and 61.8% against all laboratory-confirmed pertussis); vaccine efficacy was 42.4% and 5.7%, respectively, with the investigational 2-component vaccine that was never marketed and 28.5% and 3.1% with the wP vaccine [52]. In this same study, vaccine efficacy against pertussis with paroxysmal cough was >70% when levels of FIM2/3 antibodies were high (>5 enzyme-linked immunosorbent assay [ELISA] units [EU]) compared with an efficacy of 46.1% when antibodies to PT were high, indicating that anti-FIM antibody may provide a possible correlate of protection against pertussis [52]. In a study in Swedish infants who received a 3-dose primary series, efficacy against culture-confirmed pertussis 1 month after dose 3 was 85.2% with a 5-component aP vaccine, 58.9% with a 2-component aP vaccine and 48.3% with a wP vaccine (Table 1) [14]. In another study in Swedish infants vaccinated with a 3- and 5-component aP vaccine, less protection against mild pertussis (all culture-confirmed cases, irrespective of cough) was achieved with a 3-component vaccine compared with a 5-component vaccine containing FIM2/3 (relative risk, 1.82 [1.14, 2.90]), but no difference was observed for culture-confirmed pertussis with paroxysmal cough ≥21 days [41]. Antibodies against FIM2/3 are specific and do not cross-react. However, strains that express predominantly FIM2 can also express FIM3 during infection [47], suggesting that antibodies to both antigens may be important for optimal protection. FIM2/3 may play a role in maintaining durable immunity against pertussis. In infants receiving a 2 + 1 series of a 5-component aP vaccine, the percentage of children with antibodies above the lower limit of quantitation at 3–4 years postvaccination was higher for FIM2/3 (94.4%) compared with PT (58.4%), FHA (80.9%) and PRN (66.1%) [53].

A number of effectiveness studies support the use of 5-component aP vaccines. In Canada, the national decline in pertussis among preschool and school-aged children after 1998 has been attributed to the effectiveness of the 5-component aP vaccine deployed in 1997 [54]. An effectiveness study conducted in infants in the UK found that a 5-component aP vaccine performed as well as a highly effective wP vaccine [55] and a low-dose, 5-component aP vaccine used in the UK to immunize during pregnancy provided >90% effectiveness against laboratory-confirmed pertussis in infants <3 months of age during the 3 years following introduction [20]. Real-world effectiveness data support vaccines with single, as well as multiple pertussis components, which is not completely aligned with clinical efficacy study findings. In addition, based on available data of aP vaccine effectiveness, the WHO concluded that significant differences among pertussis vaccines based on antigen number cannot be confirmed [7]. The discrepancy between clinical trial efficacy and real-world effectiveness data underscores the need for caution when applying the results of clinical trials to diverse countries with different vaccination programs and target populations, as effectiveness is influenced by a number of factors such as vaccination schedule, adherence and herd immunity—all of which can vary substantially outside of a controlled study.

## 3. Value of Multiple Antigens in Acellular Vaccines

The inclusion of multiple and diverse pertussis vaccine antigens is expected to induce a broader immune response compared with 1-component vaccines that target only PT. The clinical value of antibodies targeting an array of bacterial antigens is under investigation and it is currently unclear which bacterial targets may offer the best clinical advantage. Opsonizing antibody against pertussis is not often assessed in clinical trials but may be more indicative of protection compared with immunoglobulin (Ig) quantity or isotype. In a study that examined paired sera from symptomatic and convalescent patients (median of 3 years after disease), antibodies to FHA and to a lesser extent PT, were the most efficient for inducing opsonization by neutrophils [56].

**Figure 1:**
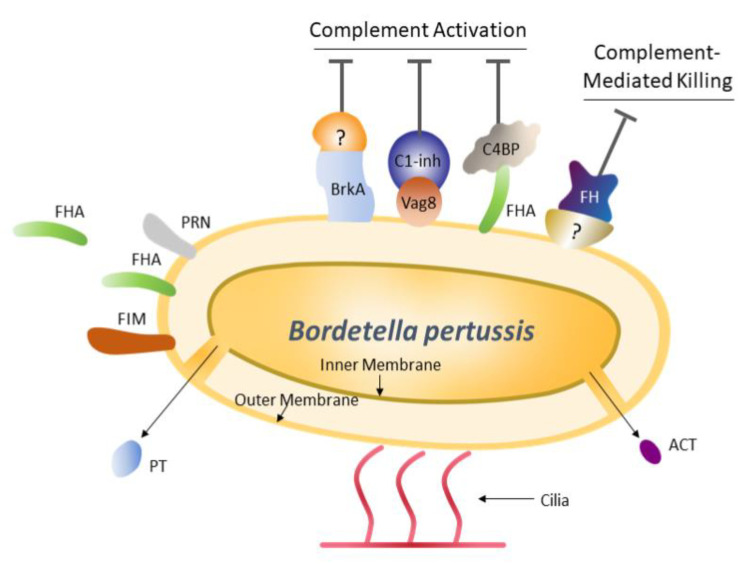
Immunological responses to *Bordetella pertussis* infection.

*B. pertussis* has evolved immune evasion strategies that facilitate survival in the human host, such as circumventing complement activation and complement-mediated killing. In addition to directly killing bacterial pathogens, complement may regulate T-cell responses, either directly or through antigen-presenting cells, making this system important for adaptive immunity [57]. The bacterium has a number of surface receptors that bind to and sequester key components of the complement pathway (Figure 1) [57]. For example, FHA binds the complement regulator C4b-binding protein—a modulator of the complement activation cascade—and tethers it to the bacterial surface, which results in protection of the bacterium from complement-mediated phagocytosis [58]. By eliciting antibodies against key bacterial receptors, such as FHA, vaccination offers the potential to disrupt receptor-ligand interactions that contribute to the diminution of complement activity.

Inhibition of complement activation occurs through the binding of bacterial surface receptors (BrkA, Vag8 and FHA) to ligands involved in the complement cascade (an unknown ligand, C1-inh and C4BP, respectively). An unknown bacterial receptor binds FH and FH-like proteins to inhibit complement-mediated killing [57].

ACT, adenylate cyclase toxin; BrkA, *Bordetella* resistance to killing protein A; C1-inh, C1 inhibitor; C4BP, C4b-binding protein; FH, factor H; FHA, filamentous hemagglutinin; FIM, fimbriae; PRN, pertactin; PT, pertussis toxin; Vag8, virulence associated gene 8.

## 4. Potential Correlates of Protection for Pertussis

The lack of universally accepted immunological correlates of protection has hampered the assessment of aP vaccine efficacy in clinical trials that use end points such as geometric mean concentrations, geometric mean fold rise and percentage of responders. Despite this, several studies report antibody associated with protection [52,59,60]. In children with household exposure to pertussis, the highest efficacy (~70%) against pertussis occurred when antibodies to FIM2/3 or PRN were high (≥5 IgG EU) versus low (<5 IgG EU), and increased even further (~85%) when both FIM2/3 and PRN antibody levels were high [52]. This model has since been validated and extended to predict efficacy based on the number of vaccine doses [59]. For vaccination with a 5-component aP vaccine, efficacy was estimated to be 82–83% after dose 3, 74–75% before booster dosing and 83–84% after booster dosing [59]. In another study of children with household exposure to pertussis that examined PT as a sole correlate of protection, significant correlations were found between lower PT antibody levels (79 U/mL) and severe disease and between higher levels (246 U/mL) and protection; a similar pattern was seen in children without household exposure [60]. In these studies, antibodies were quantitated using ELISA, and the reagents and procedures can deviate between laboratories. Therefore, establishing universally accepted, standardized correlates of protection will be important for evaluating new vaccines, and it is likely that multiple correlates of protection will be needed, given the complexity and interrelatedness of immunity to pertussis [61]. One shortcoming of the current models is the lack of consideration for cell-mediated immunity, which plays an important role in protection against pertussis. In a baboon challenge model, IgG antibodies correlated with protection against disease; however, a robust T-cell response, particularly a T-helper (TH)1/TH17 response, led to a reduction in carriage and transmission, underscoring the importance of priming for the correct immune response with vaccination [62].

## 5. Genetic Changes Attributed to Vaccines

Independent genetic changes, from single nucleotide polymorphisms to complete gene loss, have been observed in *B. pertussis* antigens, with mutations increasing over time, as discussed below. Although there is no present indication that the effectiveness of current aP vaccines has been compromised due to bacterial mutations, continued surveillance is needed.

### 5.1. Pertactin Loss

In the US and Australia, both of which have high aP vaccine coverage, most circulating *B. pertussis* strains are PRN negative [2,40]. PRN loss is also increasing in Europe, including in Denmark where a 1-component aP vaccine (containing PT only) is in use [63]. Loss of PRN is caused by functional inactivation of the gene by several independent mutations. In recent isolates from the US, those mutations include promoter disruption, deletion of the signal peptide region, 3 different C-to-T nonsense mutations leading to premature stop codons and gene disruption by an insertion sequence element [64]. The occurrence of multiple independent mutations, along with the dramatic rise in the proportion of strains lacking PRN, suggests selection against PRN. In this regard, aP vaccines have been purported to select for PRN loss [38]; however, this theory does not account for loss of this gene in Denmark, where the vaccine lacks PRN [63]. Loss of PRN as an immunological target has not diminished the efficacy of aP vaccines [65] but places additional importance on achieving protective antibodies against vaccine antigens that can confer protection, such as FIM2/3.

### 5.2. Pertussis Toxin Overexpression

A shift in PT promoter alleles from *ptxP1* to *ptxP3* beginning in 1991 yielded strains of *B. pertussis* with increased PT production and virulence [66]. Across a group of 12 European countries, *ptxP3* allele prevalence increased from 57% in 1998–2001 to 87% in 2002–2006 and 97% in 2007–2012 [67]. In the Netherlands, utilization of *ptxP3* has been linked to pertussis resurgence. In 1989, *ptxP3* accounted for 0% of isolates, increasing to 100% by 2004. The *ptxP3* promoter lineage also increased expression of additional virulence factors, including virulence associated gene 8 that is involved in complement resistance [68], and decreased expression of PRN [66].

## 6. Novel Vaccine Strategies

Acellular vaccines are protective against pertussis disease in both clinical studies and real-world settings but have been less protective against colonization and transmission than wP vaccines and natural infection. This scenario was demonstrated in a baboon model in which vaccination with a 5-component aP vaccine was protective against the symptoms of disease, but did not prevent colonization or speed clearance time compared with unvaccinated controls [62]. In this model, previous infection completely protected against both disease and colonization. aP-vaccinated baboons were also able to infect naïve controls, demonstrating that transmission was not interrupted. Robust IgG antibodies to all 5 vaccine antigens were observed after aP vaccination, suggesting a lack of correlation between the magnitude of the IgG response and colonization and transmission. T-cell responses after aP vaccination were predominantly TH2 with a weak TH1 response, while animals that were previously infected, and thereby protected, had a strong TH17/TH1 response [62]. Given the importance of cell-mediated immunity in disease protection at the population level (herd immunity), novel vaccine strategies that target the appropriate immune responses are needed. Because *B. pertussis* targets respiratory epithelia, mucosal (IgA) immunity may be a key factor for disease prevention. With a better understanding of pertussis pathophysiology, novel prophylactic approaches are being evaluated (Table 2 and summarized below).

### 6.1. Adenylate Cyclase Toxin

Adenylate cyclase toxin (ACT) is a virulence factor of *B. pertussis* that induces disruption of cell signaling leading to pathogenesis [21]. In mice, ACT did not confer protection when administered alone, but a modified ACT toxin lacking adenylate cyclase activity administered with a 2-component aP vaccine significantly reduced bacterial colonization after internasal challenge compared with aP vaccine alone [75]. Modified ACT also increased IgG2a antibodies directed against PRN and bolstered both TH1 and TH2 responses [75]. In humans, primary pertussis infection induces a strong immune response to ACT, but a blunted response was observed in aP vaccine failures [78], which may limit the utility of new vaccine strategies that include ACT.

### 6.2. Outer Membrane Vesicles

Outer membrane vesicles (OMV) are nonreplicative, spherical proteoliposome vesicles derived from bacterial membranes that contain an array of bacterial proteins in their native configurations. OMVs can be engineered to express heterologous antigens, either on exposed surfaces to drive B-cell responses or within the lumen to drive T-cell responses [79,80]. OMVs are highly immunogenic and may generate more robust and longer-lasting immunity compared with aP vaccines [81]. Efficacy of a *B. pertussis* OMV vaccine was compared with a 3-component aP vaccine in mice challenged with PRN-positive and PRN-negative strains [76]. Bacterial lung colonization by both strains was significantly reduced by the OMV vaccine compared with the aP vaccine, and the OMV vaccine increased IL-17, indicative of a TH17/TH1 response, and CD4 T cells with a tissue-resident memory phenotype [76].

### 6.3. Novel Adjuvants

Next-generation adjuvants could improve the efficacy of current aP vaccines, and numerous preclinical studies are underway in mice using traditional alum-absorbed aP vaccines delivered in conjunction with TH17/TH1-promoting adjuvants. One group of adjuvants under investigation are agonists for Toll-like receptors (TLRs), which are cell surface receptors that recognize pathogen-associated molecular patterns; binding and activating these receptors can boost humoral immunity and/or drive a TH17/TH1 response [69,82]. In mice, an alum-absorbed TLR7 agonist adjuvant together with a 3-component pertussis vaccine elicited an increased IgG response compared with the aP vaccine control and induced a TH17/TH1 response upon challenge; the control aP vaccine induced a TH17/TH2 response [69]. Similar findings were observed with a vaccine containing a TLR2 agonist combined with cyclic dimeric guanosine monophosphate—an intracellular receptor stimulator of interferon genes—which promoted a TH17/TH1 response and was protective against nasal colonization [70]. Shifting towards a TH17/TH1 response was also accomplished by inclusion of *Bordetella* colonization factor A as an adjuvant in a 3-component aP vaccine, with improved bacterial clearance from the lungs compared with the unadjuvanted control [71]. In mice, intranasal administration of an aP vaccine formulated with the adjuvant curdlan, a 1,3-β-glucan polysaccharide, induced mucosal IgA and serum IgG responses [72]. Curdlan binds to dendritic cells through the ligand Dectin-1, leading to a nuclear factor κB-mediated TH17 immune response. Additionally, curdlan forms a gel at neutral pH, helping to localize the vaccine in the respiratory epithelium leading to higher local availability [72,83]. The addition of adjuvants that enhance the immune response offer the possibility of inducing better immunity in aP vaccines that have an established safety and efficacy profile.

### 6.4. Live Attenuated, Nasally Administered Vaccines

Given that pertussis is acquired through the respiratory tract, nasally administered vaccines that stimulate mucosal immunity may prove more efficacious than vaccines eliciting systemic immunity. A live attenuated pertussis vaccine (*B. pertussis* strain BPZE1) lacking activity of dermonecrotic toxin and tracheal cytotoxin was developed and is currently in clinical trials (NCT03942406 and NCT03541499). Because of the pertussis strain used for development, this vaccine also lacks FIM3. In a preclinical study in juvenile baboons, a single dose of BPZE1 induced both systemic IgG and mucosal IgA responses and prevented disease after high-dose challenge; importantly, nasopharyngeal colonization after challenge was significantly reduced compared with unvaccinated controls, suggesting that this vaccine may disrupt colonization and transmission [73]. A phase 1 dose-finding study of BPZE1 has been completed in 48 healthy adult participants [74]. Across low-, medium- and high-dose groups, antibodies against PT, FHA, PRN and FIM2 were detected between days 14–28 and remained high at six months. However, colonization rates with BZPE1 were low (7 of 48 participants) and there was a strong association between nasal colonization with BPZE1 and antibody response, with antibodies elicited only in colonized participants in a dose-independent fashion. Mucosal IgA response was not assessed in this study [74]. A more recent iteration of BPZE1 has been developed that contains FIM3 (BPZE1f3), and antibodies to both FIM2 and FIM3 were detected in mice [84]. A second live intranasal pertussis vaccine (GamLPV) is currently in phase 1/2 clinical trials (NCT04036526).

Another approach to a live vaccine is based on the concept of disrupting the expression of immunomodulators. A strain of *Bordetella bronchiseptica*, lacking a regulator of immunomodulators (*btrS*), provided completely sterilizing immunity to all of the classical Bordetellae (*B. pertussis, Bordetella parapertussis* and *B. bronchiseptica*), demonstrating that it is possible to induce better protection than convalescent immunity [77]. Utilizing this *btrS*-deficient strain, or a simliar *B. pertussis* mutant strain, along with the understanding gained from functional studies, should make it possible to substantially improve vaccine efficacy. Although at an early stage of investigation, this approach also has the potential to both improve and broaden protective immunity to other species not covered by current vaccines.

## 7. Regulatory Considerations for Novel Acellular Vaccines

Considerable time will be needed to create a novel vaccine backbone that encompasses the virtues of low reactogenicity characteristic of aP vaccines and appropriate cell-mediated immunity characteristic of wP vaccines. While new strategies are clearly needed, regulatory challenges have the potential to forestall advances in vaccine development. Although bridging studies have aided in the timely approval of higher valency vaccines comprising the five common aP antigens, this pathway is not available for new vaccine components, which will require the full compendium of clinical data with peer review. Similar considerations are applicable for novel adjuvants. For vaccines with new components, timely approval will require cooperation between scientific development teams and regulators to leverage the knowledge obtained through previous investigational studies and real-world evidence. In the meantime, current pertussis vaccines provide protection from disease and have a good safety profile. These vaccines should be used according to established vaccination schedules, including booster dosing, with attention directed at increased compliance.

## 8. Conclusions

In conclusion, endemic disease coupled with periodic outbreaks of pertussis warrant continued vaccination diligence, as reduced coverage could lead to an overall increase in incidence. The antigens included in current aP vaccines provide biologically relevant immunological targets and have a record of safety and effectiveness. Maximizing use of currently available aP vaccines is essential for global health while next-generation vaccines are in development.

## Figures and Tables

**Table 1:** Vaccine effectiveness reported in clinical studies of acellular pertussis vaccines.

	2-Component aP ^a^		5-Component aP		wP	
	VE, %	95% CI	VE, %	95% CI	VE, %	95% CI
Culture-confirmed pertussis with ≥21 days of paroxysmal cough						
Storsaeter 1998 [52]	42.4	19.9, 58.5	75.4	59.2, 85.2	28.5	1.6, 48.0
Gustafsson 1996 [14]	58.9	50.9, 65.9	85.2	80.6, 88.8	48.3	37.0, 57.6
≥1 day of paroxysmal cough and positive laboratory criteria						
Storsaeter 1998 [52]	5.7	−9.1, 19.6	61.8	47.4, 72.2	3.1	−12.9, 16.8

^a^ Vaccine was investigational and was not marketed. aP, acellular pertussis; VE, vaccine efficacy; wP, whole-cell pertussis.

**Table 2:** Novel adjuvants and vaccines for *Bordetella Pertussis*.

**Novel Adjuvants**			
**Studied In**	**Adjuvant**	**Effect**	**Immune Response**
Mice [69]	Alum-absorbed TLR7a agonist	Higher PT neutralizing antibodies and increased inhibition of FHA binding to lung epithelium	Induced TH1/TH17 response and IgG2a/b
Mice [70]	Cyclic dimeric guanosine monophosphate with a *B. pertussis* TLR2 agonist	Combined effect of intracellular induction of interferon genes and broader TLR stimulation	Induced IFN-β, IL-12 and IL-23 and maturation of dendritic cells
Mice [71]	*B. bronchiseptica* colonization factor added to alum	Improved clearance of *B. pertussis*	Induced TH1/TH17 response and IL-17
Mice [72]	Curdlan (1,3-β-glucan)	“Sticky” properties promote vaccine localization; binds to dendritic cells and induces NF-κB	Increased IL-17; intranasal mucosal IgA and serum IgG response
**Novel Vaccines**			
**Studied In**	**Target/Strategy**	**Effect**	**Immune Response**
Non-human primates; humans [73,74]	Live attenuated intranasal *B. pertussis* BPZE1 ^a^	Reduced nasopharyngeal colonization	Induced serum IgA and systemic IgG response
Mice [75]	Detoxified ACT combined with 3-component aP vaccine	Reduced bacterial counts in lungs postchallenge	Induced IgG2a response and stronger TH1 and TH2 response
Mice [76]	*B. pertussis* OMV	Induced lung tissue-resident memory cells and reduced bacterial counts in lungs	Increased IL-17 levels
Mice [77]	*btrS-*deficient strain of *B. bronchiseptica*	Induced broad cross-species protection against *B. pertussis*, *B. parapertussis* and *B. bronchiseptica*	Increased immunity by disrupting bacterial suppression of host immune responses

^a^ Lacking dermonecrotic toxin and tracheal cytotoxin; PT was genetically detoxified. ACT, adenylate cyclase toxin; aP, acellular pertussis; *btrS*, a BvgAS-regulated extracytoplasmic function sigma factor; IFN, interferon; Ig, immunoglobulin; IL, interleukin; NF-κB, nuclear factor kappa B; OMV, outer membrane vesicles; PT, pertussis toxin; TH, T helper; TLR, toll-like receptor.
